# Draft Genome Sequence of a Strain of Cosmopolitan Fungus *Trichoderma atroviride*

**DOI:** 10.1128/genomeA.00287-15

**Published:** 2015-05-07

**Authors:** Xiaoqian Shi-Kunne, Michael F. Seidl, Luigi Faino, Bart P. H. J. Thomma

**Affiliations:** Laboratory of Phytopathology, Wageningen University, Wageningen, the Netherlands

## Abstract

An unknown fungus has been isolated as a contaminant of *in vitro*-grown fungal cultures. In an attempt to identify the contamination, we isolated the causal agent and performed whole-genome sequencing. BLAST analysis of the internal transcribed spacer (ITS) sequence against the NCBI database showed 100% identity to *Trichoderma atroviride*, and further alignment of the genome assembly confirmed the unknown fungus to be *T. atroviride*. Here, we report the draft genome sequence of a *T. atroviride* strain.

## GENOME ANNOUNCEMENT

Fungal and bacterial contamination is a widespread problem during *in vitro* cultivation practices. Recently, we experienced fungal contamination that frequently overgrew our cultures of the fungal plant pathogen *Verticillium dahliae* during routine lab cultivation on potato dextrose agar medium. In an attempt to characterize this fungal contaminant, we isolated the strain and performed whole-genome sequencing.

Using an Illumina platform, we sequenced a mate-pair library (150-bp read length, 5-kb insert size), generating 17.5 million reads in total. We subsequently assembled the genome using the A5 pipeline (1), and the remaining sequence gaps were subsequently filled using SOAP GapCloser (2). The size of the assembled draft genome is 36.4 Mb, with a G+C content of 49.7%. The assembly comprises 59 scaffolds (≥1,000 bp) and 357 contigs (>0 bp), with a scaffold *N*_50_ value of 2.1 Mb. We subsequently assessed the completeness of the assembled gene space using the CEGMA pipeline to identify orthologs of 248 core eukaryotic gene families (3), showing that 94.0% of the 248 core genes are present in our draft genome assembly. Next, repetitive elements in the genome assembly were identified with RepeatMasker (4) (http://www.repeatmasker.org) based on known repetitive elements and on *de novo* repeat identification. In total, 3.4% of the genomic DNA can be classified as repeats. The protein-coding genes in the genome assembly were annotated with the Maker2 pipeline (5), utilizing 35 predicted fungal proteomes to guide gene annotations, as described previously (6), identifying 9,127 protein-coding genes*.*

In order to reveal the identity of the fungal contamination, we first identified the internal transcribed spacer (ITS) sequence in the assembly. Nucleotide BLAST of the ITS sequence against the NCBI database resulted in 20 hits with 100% identity to various accession numbers of the ITS sequence of *Trichoderma atroviride* (top hit, GenBank accession no. ANT12-063), as well as a single hit with 100% identity to the ITS sequence of *Trichoderma harzianum* (GenBank accession no. Z48812). These were followed by a hit with 99% identity to the ITS sequence of *Trichoderma koningii* (GenBank accession no. FJ478089).

To further investigate the identity of the fungal contaminant, we used MUMmer (7) to align the assembled draft genome to the genomes of a previously sequenced *T. atroviride* strain (IMI206040) (8) and a *T. harzianum* strain (CBS226.95) available at the Joint Genomics Institute (JGI) (http://genome.jgi.doe.gov/Triha1/). The alignment showed that 92% of the *T. atroviride* genome aligns to 83% of our genome assembly, with 99.29% identity on average, whereas only 12% of the *T. harzianum* genome can be aligned to 23% of our genome assembly, with 84.98% identity on average. Thus, collectively, the ITS sequence and genome assembly alignment analysis revealed that our fungal contamination concerns *T. atroviride*. Interestingly, *T. atroviride* is one of the most common soil-borne fungal species and has been described as an antagonist to soil-borne plant pathogens (9). Therefore, *T. atroviride* is used as a biocontrol agent in agricultural settings (10). The draft genome sequence presented here complements the previously released *T. atroviride* genome sequence and will facilitate further genomic studies on the molecular biology of its biocontrol activities.

### Nucleotide sequence accession number. 

This whole-genome shotgun project has been deposited in DDBJ/EMBL/GenBank under the accession no. JZUQ00000000. The version described in this paper is the first version.

## References

[1] Tritt A, Eisen JA, Facciotti MT, Darling AE 2012 An integrated pipeline for *de novo* assembly of microbial genomes. PLoS One 7:e42304. doi:10.1371/journal.pone.0042304. 23028432 PMC3441570

[2] Luo R, Liu B, Xie Y, Li Z, Huang W, Yuan J, He G, Chen Y, Pan Q, Liu Y, Tang J, Wu G, Zhang H, Shi Y, Liu Y, Yu C, Wang B, Lu Y, Han C, Cheung DW, Yiu SM, Peng S, Xiaoqian Z, Liu G, Liao X, Li Y, Yang H, Wang J, Lam TW, Wang J 2012 SOAP*denovo*2: an empirically improved memory-efficient short-read *de novo* assembler. Gigascience 1:18. doi:10.1186/2047-217X-1-18. 23587118 PMC3626529

[3] Parra G, Bradnam K, Korf I 2007 CEGMA: a pipeline to accurately annotate core genes in eukaryotic genomes. Bioinformatics 23:1061–1067. doi:10.1093/bioinformatics/btm071. 17332020

[4] Jurka J, Kapitonov VV, Pavlicek A, Klonowski P, Kohany O, Walichiewicz J 2005 Repbase update, a database of eukaryotic repetitive elements. Cytogenet Genome Res 110:462–467. doi:10.1159/000084979. 16093699

[5] Holt C, Yandell M 2011 MAKER2: an annotation pipeline and genome-database management tool for second-generation genome projects. BMC Bioinformatics 12:491. doi:10.1186/1471-2105-12-491. 22192575 PMC3280279

[6] Seidl MF, Faino L, Shi-Kunne X, van den Berg GC, Bolton MD, Thomma BP 2015 The genome of the saprophytic fungus *Verticillium tricorpus* reveals a complex effector repertoire resembling that of its pathogenic relatives. Mol Plant Microbe Interact 28:362–373. doi:10.1094/MPMI-06-14-0173-R. 25208342

[7] Kurtz S, Phillippy A, Delcher AL, Smoot M, Shumway M, Antonescu C, Salzberg SL 2004 Versatile and open software for comparing large genomes. Genome Biol 5:R12. doi:10.1186/gb-2004-5-2-r12. 14759262 PMC395750

[8] Kubicek CP, Herrera-Estrella A, Seidl-Seiboth V, Martinez DA, Druzhinina IS, Thon M, Zeilinger S, Casas-Flores S, Horwitz BA, Mukherjee PK, Mukherjee M, Kredics L, Alcaraz LD, Aerts A, Antal Z, Atanasova L, Cervantes-Badillo MG, Challacombe J, Chertkov O, McCluskey K, Coulpier F, Deshpande N, von Dohren H, Ebbole DJ, Esquivel-Naranjo EU, Fekete E, Flipphi M, Glaser F, Gomez-Rodriguez EY, Gruber S, Han C, Henrissat B, Hermosa R, Hernandez-Onate M, Karaffa L, Kosti I, Le Crom S, Lindquist E, Lucas S, Lubeck M, Lubeck PS, Margeot A, Metz B, Misra M, Nevalainen H, Omann M, Packer N, Perrone G, Uresti-Rivera EE, Salamov A, et al. 2011 Comparative genome sequence analysis underscores mycoparasitism as the ancestral life style of *Trichoderma*. Genome Biol 12:R40. doi:10.1186/gb-2011-12-4-r40. 21501500 PMC3218866

[9] Druzhinina IS, Seidl-Seiboth V, Herrera-Estrella A, Horwitz BA, Kenerley CM, Monte E, Mukherjee PK, Zeilinger S, Grigoriev IV, Kubicek CP 2011 *Trichoderma*: the genomics of opportunistic success. Nat Rev Microbiol 9:749–759. doi:10.1038/nrmicro2637. 21921934

[10] Brunner K, Zeilinger S, Ciliento R, Woo SL, Lorito M, Kubicek CP, Mach RL 2005 Improvement of the fungal biocontrol agent *Trichoderma atroviride* to enhance both antagonism and induction of plant systemic disease resistance. Appl Environ Microbiol 71:3959–3965. doi:10.1128/AEM.71.7.3959-3965.2005. 16000810 PMC1168994

